# A randomised controlled trial and cost-effectiveness evaluation of "booster" interventions to sustain increases in physical activity in middle-aged adults in deprived urban neighbourhoods

**DOI:** 10.1186/1471-2458-10-3

**Published:** 2010-01-04

**Authors:** Daniel Hind, Emma J Scott, Robert Copeland, Jeff D Breckon, Helen Crank, Stephen J Walters, John E Brazier, Jon Nicholl, Cindy Cooper, Elizabeth Goyder

**Affiliations:** 1 Clinical Trials Research Unit, University of Sheffield, Regent Court, 30 Regent Street, Sheffield, S1 4DA, UK; 2 The Centre for Sport and Exercise Science, Faculty of Health and Wellbeing, Sheffield Hallam University, Collegiate Crescent Campus, Sheffield, S10 2BP, UK; 3 School of Health and Related Research (ScHARR), University of Sheffield, Regent Court, 30 Regent Street, Sheffield, S1 4DA, UK

## Abstract

**Background:**

Systematic reviews have identified a range of brief interventions which increase physical activity in previously sedentary people. There is an absence of evidence about whether follow up beyond three months can maintain long term physical activity. This study assesses whether it is worth providing motivational interviews, three months after giving initial advice, to those who have become more active.

**Methods/Design:**

Study candidates (n = 1500) will initially be given an interactive DVD and receive two telephone follow ups at monthly intervals checking on receipt and use of the DVD. Only those that have increased their physical activity after three months (n = 600) will be randomised into the study. These participants will receive either a "mini booster" (n = 200), "full booster" (n = 200) or no booster (n = 200). The "mini booster" consists of two telephone calls one month apart to discuss physical activity and maintenance strategies. The "full booster" consists of a face-to-face meeting with the facilitator at the same intervals. The purpose of these booster sessions is to help the individual maintain their increase in physical activity. Differences in physical activity, quality of life and costs associated with the booster interventions, will be measured three and nine months from randomisation. The research will be conducted in 20 of the most deprived neighbourhoods in Sheffield, which have large, ethnically diverse populations, high levels of economic deprivation, low levels of physical activity, poorer health and shorter life expectancy. Participants will be recruited through general practices and community groups, as well as by postal invitation, to ensure the participation of minority ethnic groups and those with lower levels of literacy. Sheffield City Council and Primary Care Trust fund a range of facilities and activities to promote physical activity and variations in access to these between neighbourhoods will make it possible to examine whether the effectiveness of the intervention is modified by access to community facilities. A one-year integrated feasibility study will confirm that recruitment targets are achievable based on a 10% sample.

**Discussion:**

The choice of study population, study interventions, brief intervention preceding the study, and outcome measure are discussed.

**Trial Registration:**

Current Controlled Trials: ISRCTN56495859; ClinicalTrials.gov: NCT00836459.

## Background

There are a number of published systematic reviews of evidence for interventions that increase physical activity [1-5]. More recently the evidence base for brief interventions in primary care has been reviewed [6]. This review identified a sufficient evidence base for NICE to recommend the use of brief interventions to promote physical activity but also identified specific evidence gaps that this trial will be able to address, particularly in relation to the value of follow up beyond three months, for the longer term maintenance of physical activity.

Searches of the National Research Register and ClinicalTrials.gov for research in progress confirm that, although there are a number of physical activity intervention trials in progress in specific patient groups and in older age groups or in children, there are few trials including "healthy" middle-aged participants and no other trials specifically examining the value of further intervention after an initially successful "brief intervention".

The Sheffield Physical Activity Booster Trial is a three-arm, parallel group, randomised controlled trial with a feasibility study. It will compare two different intensities of Motivational Interviewing (MI; 'booster') intervention against no further intervention in people who have already increased their physical activity levels following a brief intervention. The "mini booster" of two telephone physical activity consultations and a "full booster" of two face-to-face physical activity consultations will be offered four and five months after the initial brief intervention. The purpose of these booster sessions is to help participants to sustain their physical activity levels and prevent relapse. The brief intervention will involve provision of an interactive DVD based on a MI approach that is directive, client-centred and replicates the style of other successful behaviour change programmes [7,8]. All interventions, including the initial brief intervention, will be delivered by trained facilitators (employed as research assistants and trained by the research team) to ensure consistent delivery.

## Methods/Design

### Participants

We are working with local health services to identify potentially eligible study candidates. NHS Sheffield will send a letter with postage-paid reply card to at least 30,000 people to inviting them to enrol in a programme to help them get more physically active. This programme involves a "brief intervention" (interactive DVD), which is consistent with NHS guidance on physical activity and behaviour change interventions [6,9]. Research assistants will telephone respondents and administer the Scottish Physical Activity Questionnaire (SPAQ)[10]. Those eligible to receive the brief intervention (DVD) will be: (1) residents of the 20 most deprived neighbourhoods in the city of Sheffield; (2) aged 40 to 64 years; and, (3) not achieving the current recommended activity level (30 minutes of moderate activity on at least 5 days) assessed using the SPAQ and wishing to have support to become more active. Those subsequently eligible for participation in the trial will also: (4) have increased their physical activity level by at least 30 minutes of moderate or vigorous activity per week (assessed using the SPAQ) over the three-month brief intervention (DVD) period; and, (5) be capable of giving written informed consent to trial participation. Individuals with chronic conditions who can benefit from physical activity will not be excluded unless their condition significantly impairs their ability to exercise. They will be asked to consult their GP if they have a condition that increases their risk of adverse events during exercise (e.g. chronic cardiovascular or pulmonary disease). Participants may withdraw from active participation in the study on request. If a participant experiences chest pain or severe breathlessness during the 12-minute walk test (see below), then the researcher will advise the GP directly and immediately, and will also advise the participant to make an appointment with their GP at their earliest convenience. Subjects removed from active participation will not be replaced and, with their consent, will be followed up for all outcome information.

### Booster Interventions

Candidates deemed eligible after a telephone assessment will be invited to attend a baseline assessment appointment at a community venue and they will be randomly allocated to one of three groups:

1. a "full booster" group (n = 200) receiving an intervention comprising two face-to-face physical activity consultations, delivered in a motivational interviewing style, at one month and two months from randomisation;

2. a "mini booster" group (n = 200) also receiving an intervention comprising two telephone-based physical activity consultations, delivered in a motivational interviewing style, at one month and two months from randomisation; or,

3. a control group (n = 200) who will be assessed at randomisation, after three months and after nine months and receive no additional intervention between those assessments.

**Figure 1:**
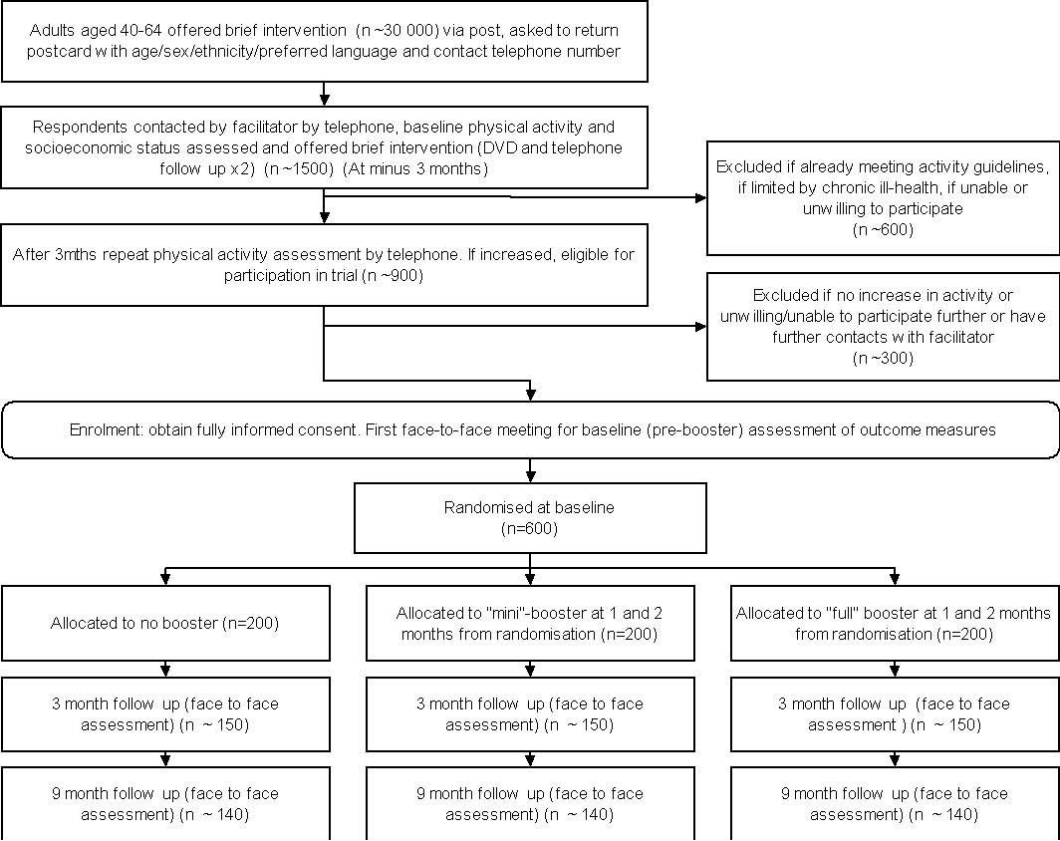
**Participant flow through the study**.

Participant flow through the study from initial contact to final follow-up is illustrated in figure 1.

The "full" booster will comprise two 20-30 minute face-to-face physical activity consultations that aim to promote and sustain change in physical activity status. These will take place in community venues. The consultation will be underpinned by the principles of the Trans-Theoretical Model (TTM) of behaviour change and replicate a brief version of motivational interviewing based on a method designed for time limited consultations in medical settings [11,12]. Such an approach has been successfully employed to change health-related behaviours previously [13]. This approach also mirrors that adopted by the health trainer initiative which provides a current model of face-to-face promotion of healthy behaviours. For the "full" booster, a menu of six strategies has been developed to guide the 30-minute consultation. Each strategy is suitable for participants who are in the maintenance stage of motivational readiness for physical activity behaviour change. They are: 1. assessment of motivation and confidence for maintaining physical activity; 2. increasing knowledge of the benefits of physical activity and the risks of a sedentary lifestyle, and increasing awareness of physical activity opportunities and the current recommendations for physical activity; 3. increasing confidence to be physically active - self-efficacy; 4. goal setting and tracking using SMART (specific, measurable, achievable, realistic, time-related) goal principles; 5. strategies for staying motivated; and, 6. relapse-prevention strategies. During the "full" booster consultations, strategies will be worked through at a pace dictated by the participant and the menu used to structure information exchange without being prescriptive.

Although face-to-face interventions have been found to be efficacious in promoting physical activity, many of the barriers associated with this approach, including time and financial costs, highlight the need for pragmatic alternatives that are both relatively cheap to deliver and may make it easier for the participant to access the intervention [13]. The "mini" booster will consist of two 20-30 minute telephone based physical activity consultations. The telephone consultation will follow the same menu of six behaviour change strategies as the "full" booster (outlined above) and aim to promote and sustain change in physical activity status. A number of studies using telephone support for physical activity in older adults have been carried out [3,14]. A telephone-based approach has been effective in increasing physical activity participation at six months compared to no telephone support and has also been shown to increase physical activity participation to a greater extent than standard reading materials in adult populations [14,15]. The telephone consultations will follow a script of known efficacy that has been implemented in previous physical activity promotion studies delivered by members of this research team.

The MI training and interventions will follow a treatment fidelity framework, based on the Behaviour Change Consortium, to ensure reliability and consistency of the approach [16,17]. This framework provides quality assurance parameters based on the intervention design, training, delivery, receipt and enactment. A recent review [16,17] highlighted inconsistent delivery and varying levels of competence among physical activity interventionists reporting to deliver a physical activity counselling components. It suggested that the effectiveness of behaviour change counselling is predicted by the length, the intensity, the content of interventions and the competence of the deliverer [17]. The sessions will therefore be delivered by a team of six research assistants (RAs) trained in MI and behaviour change techniques and assessed to ensure their competency. A framework will be developed for each session to ensure consistency of advice across sessions and between participants. All RAs will be trained (by JDB, HC and RC) using a training package and a detailed manual to ensure standardised delivery of the booster interventions. All booster interventions will be video-recorded. A random selection of 5% of all booster consultations (10 telephone and 10 face-to-face) will be reviewed and assessed by an independent clinical psychologist using a pre-determined check list. The RAs will be provided with individual feedback if required, to ensure intervention fidelity is maintained.

### Objectives

The overall aim is to measure the effectiveness and cost effectiveness of "mini" and "full" booster sessions, as an adjunct to a brief intervention, in sustaining physical activity in middle-aged adults. The primary objective is to determine whether physical activity, measured by accelerometry three months after randomisation (six months after a brief intervention), is significantly increased in participants allocated to two intervention groups ("full" or "mini" booster) compared to participants allocated to a control group (receiving no further contact after the baseline assessment). Secondary objectives are as follows:

1. To determine whether physical activity nine months after randomisation (12 months after the brief intervention) is significantly increased in participants allocated to the two intervention groups compared to participants allocated to the control group.

2. To compare physiological measures of fitness (12 minute walk test) and self-reported physical activity (SPAQ) between allocated groups.

3. To compare health related quality of life, resource use (including health and social care contacts) and economic costs between allocated groups.

4. To investigate whether the impact of the intervention may be modified by gender, ethnicity or the types of physical activity undertaken (including use of community facilities for physical activity).

5. To undertake a process evaluation to identify, using both quantitative and qualitative methods, psychosocial and environmental factors that may mediate or modify the effectiveness of the intervention (this aspect of the study is the subject of a separate protocol and is not discussed further here).

### Outcomes

The timing of all outcomes relative to the point of randomisation is shown in Table 1. The primary outcome is seven-day accelerometry, using Actiheart (CamNtech, Cambridge, UK), measured at three months and nine months post-randomisation (see Discussion). Secondary outcomes are:

**Table 1:** Timing of procedures and assessments

	Minus 3 months	Minus 2 months	Minus 1 month	~Minus 1 week	Randomisation	1 month	2 months	3 months	9 months
Brief Intervention Screening Checklist	✔								
SPAQ	✔			✔				✔	✔
DVD (if eligible)	✔								
DVD usage assessment		✔	✔						
Booster Trial Screening Checklist				✔					
Participant Information Sheet				✔	✔				
Participant Consent Form					✔				
BREQ-2					✔			✔	✔
EXERT					✔			✔	✔
Questionnaire 1					✔				
Questionnaire 2					✔			✔	✔
Questionnaire 3 (SF-12v2+4)					✔			✔	✔
Height & weight					✔			✔	✔
Randomisation					✔				
Booster intervention (Rx only)						✔	✔		
12 minute walk test								✔	✔
7 day accelerometery								✔	✔

1. Self-reported moderate or vigorous physical activity using the Scottish Physical Activity Questionnaire (SPAQ) which records type and duration of activities in the previous week;

2. Behavioural Regulation in Exercise Questionnaire (BREQ-2) [18];

3. Exercise Evaluation Randomised Trial (EXERT) questionnaire [19];

4. Health-related quality of life using the Sheffield Version of the 16-item Short Form health survey instrument (SF-12v2 plus 4);

5. Self-reported use of community facilities for physical activity;

6. Self-reported health and social care contacts;

7. Body mass index; and,

8. The 12 minute walk test (physiological measures of fitness) [20].

### Sample size

For the purposes of sample size estimation the primary outcome is an objective measure of physical activity and will be the mean physical activity levels from the 7-day accelerometric assessment (recoded as counts per week) at 3 months post-randomisation (6 months after initial contact). Kirk and colleagues suggest a standard deviation of approximately 1.2 million counts per week from a 7-day accelerometer assessment of 70 patients with type II diabetes [21]. We assume a mean difference of 400,000 counts per week between the intervention and control groups is the smallest difference of clinical and practical importance that is worth detecting. With 450 subjects (300 intervention: 150 control), the trial will have 90% power to detect this mean difference or greater between the "booster" arm and control arm (assuming a standard deviation of 1.2 million counts/week) as statistically significant at the 5% (two-sided) level using a two independent samples t-test.

With 300 subjects in the booster intervention (150 "mini": 150 "full" booster) the trial will have also have approximately 80% power to detect a similar mean difference of 400,000 counts per week between the two booster arms as statistically significant at the 5% (two-sided) level using a two independent samples t-test. Assuming an approximate 25-30% loss to follow-up by six-months, we proposed to recruit and randomise 200 subjects per group (600 in total). We will invite at least 30,000 40-64 year olds from the 20 most deprived neighbourhoods; we expect at least 1500 will receive the brief intervention of which we anticipate at least 40% will increase their physical activity and be eligible for randomisation. N = 600 divided equally between the three trial arms (200 per arm).

### Randomization sequence generation

The allocation schedule is generated prior to the study by the Sheffield Clinical Trials Research Unit. The randomisation sequence is computer-generated.

### Allocation concealment

The allocation schedule will be concealed through the use of a centralised web-based randomisation service.

### Implementation

During the baseline visit, after informed consent has been obtained, the research assistant will telephone the study manager or data manager, who will randomise the participant. The research assistant will inform the participant of the treatment allocation and the study team will inform their general practitioner that they have been recruited to the study.

### Blinding

Data analysts will be blind to treatment allocation, but the study manager, research assistants and participants (who are also outcome assessors) will not be blinded.

### Ethical approval

This study was reviewed and approved by Sheffield Research Ethics Committee.

### Statistical methods

The primary analysis will be of accelerometry data at three months post-randomisation. Analysis will be by intention-to-treat. A sensitivity analysis will be undertaken to impute missing accelerometry data using baseline and follow-up data from the group of participants with valid accelerometry data at three months post-randomisation. As this is an ITT analysis, withdrawals and protocol violations will be analysed in their groups as randomised.

All statistical exploratory tests will be two-tailed with alpha = 0.05. Baseline demographic variables (age, gender), physical measurements (e.g. weight, height, BMI), and health-related quality of life data (SF-36) will be summarised with appropriate summary statistics, tabulated and assessed for comparability between the treatment groups. For example, categorical variables, e.g. gender, the number and percentage of each category (e.g. male and female) will be reported. For continuous variables, e.g. age, reporting will depend on the distribution of the data, if it is symmetric, the data will be summarised with a mean and standard deviation; if it has a non-symmetric distribution it will be summarised with a median and inter-quartile range.

The primary aim is to compare the intervention ("full" or "mini" booster) versus control treatment (No booster), with a secondary aim of comparing the two interventions ("full" versus "mini" booster). The primary comparison will be between the mean physical activity levels from the accelerometer (counts per week) in the two "booster" arms combined compared with the mean physical activity levels in the control arm at 6 months follow-up (3 months post-randomisation). This difference in means, between the intervention and control groups, will be compared using a two independent samples t-test and a 95% confidence interval for estimated mean difference between the groups will also be calculated. In the event of differences between the booster and control groups with respect to baseline demographic, physical, and health-related quality of life measurements, multiple regression will be used to adjust the treatment effect for these variables. The ordinary least squares adjusted regression coefficient estimate for the treatment group parameter along with its 95% confidence interval (CI) will then be reported.

The research hypothesis is that the booster intervention groups will have greater levels of physical activity than the control group. The statistical and null hypothesis is that there are no differences between the intervention and control groups at follow up. The alternative hypothesis is that there is a difference in physical activity levels between the intervention and control groups at follow up.

Secondary aims are to compare the effect of the two interventions ("full" versus "mini" booster). This will be done using the same methods as for the primary endpoint as described above. Interim analyses will not be required. An exploratory sub-group analysis using multiple linear regression with the primary outcome, the mean physical activity levels from the accelerometer (counts per week) at 6 months follow-up, will look for an interaction between treatment group (booster or control) and sub-groups defined by gender, ethnicity and access to community facilities (self reported use versus no use of community facilities).

Analyses of secondary outcomes will identify any significant different between groups for each outcome measure, at three months and nine months from randomisation:

1. Physiological measures of fitness (12 minute walk test) and types of physical activity (self report) and change in self-reported physical activity levels

2. Change in health-related quality of life measured by changes in SF-12v2 plus 4 (converted to SF-6D)

3. Health and social care contacts

4. Changes in psychological measures of motivation, intention and stages of change, and self-efficacy

Secondary categorical outcomes such as the proportions maintaining (or increasing) their weekly duration of physical activity in the two "booster" arms combined compared with the proportion in the control arm at 6 months follow-up (3 months post-randomisation), will be compared between the intervention and control groups, using a continuity corrected chi squared test and a 95% confidence interval for estimated differences in proportions will also be calculated. In the event of differences between the groups with respect to baseline demographic, physical, and health-related quality of life measurements, multiple logistic regression will be used to adjust the treatment effect for these variables. The maximum likelihood estimated regression coefficient for the treatment group parameter (odds ratio) along with its 95% confidence interval (CI) will then be reported.

Secondary outcomes such as HRQoL (SF-12v2 plus 4 dimension scores) and distance walked on 12 minute walk test, at three months post-randomisation, will be assumed to be continuous outcomes. A two independent samples t-test will be used to compare mean outcomes between the booster and control groups in this parameter. A 95% confidence interval (CI) for the mean difference in this parameter between the groups will also be calculated. In the event of differences between the booster and control groups with respect to baseline demographic, physical, and health-related quality of life measurements, multiple regression or analysis of covariance (ANCOVA) will be used to adjust the treatment effect for these variables. The ordinary least squares adjusted regression coefficient estimate for the treatment group parameter along with its 95% confidence interval (CI) will then be reported. Outcomes assessed at nine months post-randomisation will be analysed in a similar way. We shall also compare the effect of the two interventions ("full" versus "mini" booster) on these secondary outcomes at three and nine months post-randomisation, using the same methods as described above.

The health economic component of the study will estimate the incremental cost effectiveness of the "mini" booster and full booster interventions compared to no booster. It will include an estimation of the cost effectiveness of the intervention from a NHS perspective in terms of their incremental cost per quality adjusted life year (QALY) and a broader societal assessment of efficiency that includes costs for other Government agencies and productivity (inside and outside the home). It uses similar methods to those used in the successfully completed evaluation of a community exercise programme [22].

There will be two components to the costing. The interventions will be costed, as well as the consequences for the use of health and social services in general. The costs of the booster consultations will be assessed in a micro costing study. The costs will include enrolment of participants, training and time of facilitators, travel and telephone calls. Actual cost data will be collected for consumables and facilitator time will be costed using national grades. Despite being a highly pragmatic trial, there are some features of the programme which are specific to the research study and it will be necessary to adjust for these in order to make the results generalisable. Care will also be taken to compare costs assuming a routine level of throughput, rather than that achieved in the trial. Any research related costs will be excluded.

The consequences for use of health and social services will use resource data collected from participants. Use of primary, secondary, community and social services will be obtained using a self-completed resource questionnaire administered to participants at each assessment at baseline, three months and nine months. Resources will be costed using the best available national estimates. Where appropriate, national unit costs will be used [23].

SF-12v2 plus 4 data will be converted into health state utility values using the SF-6D preference-based algorithm [24]. The area under the curve between assessments will be used to provide an overall estimate of the QALY difference between the intervention arms and the control arm after adjusting for significant baseline variables [25]. Given cost and benefit data will only be collected for nine months, the on-going costs and health benefits will not be discounted, though start-up costs, including training costs, will be annuitised over a five year period. The sensitivity of the results to possible uncertainties in key parameters will be explored by a full sensitivity analysis, including a probabilistic sensitivity analysis.

### Feasibility study

The main risks to trial success identified by those providing scientific review that the feasibility trial will test are:

1. Recruitment targets for the brief intervention will not be met;

2. The brief intervention will not be effective enough to generate sufficient individuals eligible for the trial;

3. Insufficient eligible individuals will consent to participate in the trial

In the first year a feasibility study will be undertaken to assess the feasibility of both trial recruitment plans and the proposed interventions [26,27]. A total of 3000 invitations will be sent to the patients of general practices situated in a "typical" deprived ward. The aim is for at least 150 people to receive the brief intervention and 60 to be randomised to the three trial arms. This will allow outcome measurement in 15-20 individuals in each study arm to estimate a mean and standard deviation for the primary outcome, physical activity counts/per week from a 7-day accelerometry assessment in each group. The success criteria for the feasibility study will be:

1. At least 60 participants recruited to the pilot trial and 45 having 3 month follow-up measurements including accelerometry completed on the basis of an initial mail-shot to 3000 individuals. (We will not use community recruitment at this stage since it may represent a more limited pool for recruitment that we can use to boost participant recruitment from "hard-to-reach" groups as required in the main trial)

2. At least 70% of those randomised to booster interventions actually receiving the interventions per protocol

3. On the basis of the pilot primary outcome (accelerometry) data collected, the sample size for the main trial will be re-calculated. The trial will not proceed if the revised sample size calculation suggests a total sample size greater than 600 will be required. Assuming the protocol and intervention remain unchanged, the participants recruited during the feasibility phase will be included in the full trial population.

## Discussion

### Choice of study population

We know that physical activity levels in the UK are low in South Asian (Pakistani, Bangladeshi and Indian) and Chinese populations with the lowest levels in the Pakistani and Bangladeshi population [28]. Previous trials of face-to-face interventions or telephone interventions have generally been limited to English speaking populations for practical reasons and there is a lack of community-based trials in deprived and in non-White populations [6]. However, meta-analysis suggests effect sizes from motivational interviewing-based interventions are large in ethnic minority populations [29]. We therefore propose to recruit from an ethnically diverse and socio-economically deprived population including Pakistani, Bangladeshi and Chinese communities by making the initial recruitment process as simple as possible i.e. the return of a postage paid reply card with basic information on preferred language and a phone number to allow a research assistant to make contact. The use of bi-lingual study partners as facilitators, where feasible, will facilitate recruitment of ethnic minority populations and allow assessment of effectiveness of the intervention in a multi-ethnic population.

### Choice of study interventions

Behavioural ("lifestyle") interventions have been shown to be potentially more cost-effective than supervised activity ("structured" interventions) and are also more easily generalisable to populations with access to different facilities [30]. There is evidence for the efficacy of telephone support, as a less expensive alternative to face-to-face physical activity consultations, including some evidence from interventions involving no face-to-face assessment or intervention [15].

Meta-analytical and systematic reviews of physical activity and behaviour change suggest that the transtheoretical model (TTM) of behaviour change is the most commonly adopted theoretical framework for promoting physical activity [31-33]. The TTM has demonstrated effectiveness as an approach to increasing exercise adoption and adherence in adults [32,34-36]. It describes how people modify problem behaviours or acquire positive new ones [11,33,37]. The TTM determines behaviour change as a process rather than a single event and offers practical suggestions for how individuals can change behaviour. It consists of the following constructs: stages of change (describes when people change), processes of change (outlines techniques for helping people to change), decisional balance (weighing up the pro's and con's of change) and self-efficacy (increasing one's confidence to change behaviour) [33]. The TTM offers practitioners a common, validated framework for guiding participants through periods of change and proposes strategies for maintaining positive behaviours. We will also adopt a client centred approach to all interventions based upon the style of motivational interviewing.

Motivational interviewing (MI) is a directive, client-centred counselling style for eliciting behaviour change by helping clients to explore and resolve ambivalence [38]. Motivational interviewing has been used in many settings and ethnic groups and has been shown to impact positively on lifestyle and health outcomes including physical activity behaviours in adults [29,39-41]. MI has been applied in a number of formats including technology-based, such as internet and video, telephone and face-to-face consultations [7,8,39,42-44]. An example of a technology-based intervention adopting an MI approach is The Drinker's Check-up [7,8,43]. The Drinker's Check-up offers a comprehensive assessment of the client's drinking and related behaviours. A key element of the programme is providing feedback that matches the user's individual circumstances, motivational readiness and confidence for changing their behaviour.

### Choice of brief intervention (DVD) preceding the study intervention

We have developed a "brief intervention" using motivational interviewing principles but in the form of an interactive DVD and telephone follow up that would be straightforward to adapt for practical application beyond the research context. Our "brief intervention" is consistent with the NICE guidance (and supporting evidence base) on physical activity and behaviour change interventions [6,9], and is based on interventions of known efficacy that are already in use in Sheffield [28]. The intervention and its development will be described more fully in a further paper.

### Choice of outcome measure

Direct measures of physical activity (rather than self-report measures) used at baseline may themselves increase physical activity. The major MRC-funded ProActive trial of physical activity promotion in people with a family history of diabetes has recently been completed and was reported at the European Diabetes Epidemiology Group 2007 meeting [45]. The investigators suggested that the negative result from this major trial may have been due to the intensive baseline measurement resulting in participants in all arms being equally successful in increasing their activity levels, since recent work suggests assessment can trigger a significant increase in activity levels [46]. There is also concern that the detailed baseline assessment usually carried out in a trial setting is a significantly more intensive intervention than is feasible in everyday practice [19]. We have therefore proposed that direct measurement of fitness and physical activity are only performed three months and nine months after randomisation.

Although the study was designed to measure activity counts per week as the objective measure of physical activity, the Actiheart devices that we are using are also capable of measuring energy expenditure, using a combination of heart rate and activity counts recorded by its two sensors. At the first Trial Steering Committee our Independent Chair suggested a change in the primary outcome, from activity counts to energy expenditure, once the feasibility phase was complete. This would capture exercise intensity as well as frequency and duration, making the study results more meaningful to policy makers as well as more comparable to recent related studies.

### Feasibility Study

A number of trials of physical activity interventions have had lower recruitment rates than predicted and have had to subsequently adapt recruitment strategies, notably both the recent MRC-funded ProActive trial and the earlier Newcastle primary care based trial [45,47]. We anticipate low recruitment rates and differential recruitment rates from different ethnic and socio-economic groups. We will therefore use multiple recruitment strategies across a large inner-city population using a very large initial mailing to ensure we reach enough potential participants from deprived communities and specific ethnic groups. If we fail to achieve planned recruitment rates in Sheffield, we will be able to roll-out recruitment in neighbouring deprived South Yorkshire communities (in Barnsley and Rotherham), as undertaken in the HTA-funded DiGEM trial of blood glucose self-monitoring, at no extra cost. Recruitment will be supported and facilitated by the regional Primary Care Network (EMSYNET). The proposed feasibility study will determine whether the proposed recruitment plans are achievable.

## Competing interests

The authors declare that they have no competing interests.

## Authors' contributions

DH and ES are the study managers who drafted the manuscript. EG is the Principal Investigator who initiated the work. RC and HC developed the protocol in conjunction with EG, who secured the funding. JDB developed the MI protocol and trained the research assistants in the approach. SJW provided advice on the study design, sample size and statistical analysis of the outcome data. JEB provided advice on health economics, cost effectiveness analysis and the related data collection tools. JN and CC consulted on the randomised controlled trial methodology. RC, HC, JDB, SJW, JEB, JN and CC were co-applicants on the funding application. All authors commented on earlier drafts of this manuscript, and read and approved the final manuscript.

## Pre-publication history

The pre-publication history for this paper can be accessed here:

http://www.biomedcentral.com/1471-2458/10/3/prepub
